# The Complete Moss Mitochondrial Genome in the Angiosperm *Amborella* Is a Chimera Derived from Two Moss Whole-Genome Transfers

**DOI:** 10.1371/journal.pone.0137532

**Published:** 2015-11-30

**Authors:** Z. Nathan Taylor, Danny W. Rice, Jeffrey D. Palmer

**Affiliations:** Department of Biology, Indiana University, Bloomington, Indiana, United States of America; Vanderbilt University, UNITED STATES

## Abstract

Sequencing of the 4-Mb mitochondrial genome of the angiosperm *Amborella trichopoda* has shown that it contains unprecedented amounts of foreign mitochondrial DNA, including four blocks of sequences that together correspond almost perfectly to one entire moss mitochondrial genome. This implies whole-genome transfer from a single moss donor but conflicts with phylogenetic results from an earlier, PCR-based study that suggested three different moss donors to *Amborella*. To resolve this conflict, we conducted an expanded set of phylogenetic analyses with respect to both moss lineages and mitochondrial loci. The moss DNA in *Amborella* was consistently placed in either of two positions, depending on the locus analyzed, as sister to the Ptychomniales or within the Hookeriales. This agrees with two of the three previously suggested donors, whereas the third is no longer supported. These results, combined with synteny analyses and other considerations, lead us to favor a model involving two successive moss-to-*Amborella* whole-genome transfers, followed by recombination that produced a single intact and chimeric moss mitochondrial genome integrated in the *Amborella* mitochondrial genome. Eight subsequent recombination events account for the state of fragmentation, rearrangement, duplication, and deletion of this chimeric moss mitochondrial genome as it currently exists in *Amborella*. Five of these events are associated with short-to-intermediate sized repeats. Two of the five probably occurred by reciprocal homologous recombination, whereas the other three probably occurred in a non-reciprocal manner via microhomology-mediated break-induced replication (MMBIR). These findings reinforce and extend recent evidence for an important role of MMBIR in plant mitochondrial DNA evolution.

## Introduction

Horizontal gene transfer (HGT) occurs surprisingly frequently between the mitochondrial genomes of land plants, especially angiosperms [1,2]. The most remarkable case involves the angiosperm *Amborella trichopoda*, otherwise best known as the singular sister group to all other flowering plants [3,4], but see [5,6]. An early, PCR-based study of *Amborella* mitochondrial DNA (mtDNA) revealed the presence of both native and foreign copies of numerous genes, with six of the foreign genes having been acquired from moss mtDNA [7]. Phylogenetic analysis of these six genes, based on limited sampling in mosses, suggested that three different mosses had each donated one of these genes and was uninformative as to the moss donors of the other three genes [7]. The orders (Ptychomniales, Hypnales, and Hookeriales) to which the three putative donors belong constitute a group referred to as the ‘homocostate pleurocarps’. Though the monophyly of the Hookeriales is sometimes debated, the current phylogeny of the homocostate pleurocarps places the Ptychomniales as sister to a clade composed of the other two orders. The homocostate pleurocarps, together with their sister order, Hypnodendrales, constitute what is known as the ‘crown pleurocarps’, which comprise about half of the ca. 10,000 species of mosses [8,9,10].

**Fig 1:**
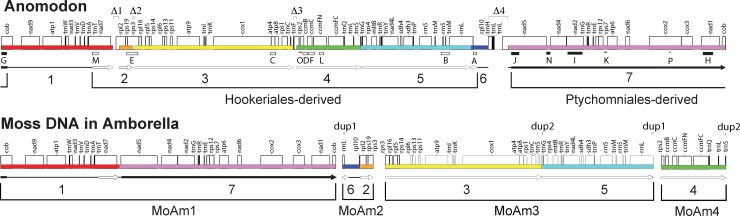
A nearly full-length moss mitochondrial genome in *Amborella* mtDNA is very similar to the *Anomodon* moss genome. **Top:** Gene map of the 104.2-kb reference mitochondrial genome of the moss *Anomodon* [13] shown linearized within the *cob* intron to correspond to a diagrammatically favorable breakpoint, of several possible ones (see Results), of a donor moss genome immediately after its integration in *Amborella* mtDNA. Colored boxes and arrows indicate the position and relative orientation, respectively, of the seven blocks of synteny between the *Anomodon* genome and the four moss-derived regions in the *Amborella* genome (see Bottom). The black arrows (or portions thereof) and the bracket labeled “Ptychomniales-derived” indicate the location relative to *Anomodon* of a region of at least 32 kb in size, part or all of which (see first section of Discussion) was acquired by *Amborella* from a Ptychomniales-like donor, while the open arrows and bracket labeled “Hookeriales”-derived indicate the location relative to *Anomodon* of a region of at least 53 kb in size, part of all or which was acquired by *Amborella* from a Hookeriales donor. The thin lines indicate the maximum extent of the three regions of unassigned origin. The 16 loci used for phylogenetic analysis are marked by rectangles or lines labeled A-P (see Fig 2 and S1 Fig and Table 1). A filled rectangle indicates a Ptychomniales-like origin, an open rectangle a Hookeriales origin, and a thin line an unresolved origin. The four deletions and two duplications >100 bp in length in *Amborella* relative to *Anomodon* are marked by “Δ1-Δ4” and “dup1” and “dup2”, respectively. **Bottom:** Syntenic arrangement of genes in the four moss-derived regions (MoAm1-MoAm4) present in *Amborella* mtDNA.

Recent sequencing of the *Amborella* mitochondrial genome revealed that it contains roughly six mitochondrial genome equivalents of DNA acquired by HGT [11]. These include two genome equivalents from angiosperms, three from green algae, and one from mosses. Most of the moss-derived protein genes present in *Amborella* are clear pseudogenes; this and other considerations suggest that few if any of the foreign moss genes are functional [11]. The foreign moss mitochondrial “genome” consists of four tracts of contiguous moss-like DNA (designated herein as “**Mo**ss-in-***Am*** *borella*” tracts MoAm1-MoAm4; see Fig 1, bottom) of lengths 48.1, 40.0, 9.3, and 4.4 kb that are located at widely separated positions in the *Amborella* mitochondrial genome [11]. These four regions are highly similar in sequence and structure to the 14 published moss mitochondrial mtDNAs [12–15]. Of greatest relevance, these 14 sequenced moss genomes are perfectly syntenic and very similar in size (all but two are between 100 and 110 kb in size). One of these genomes, the 104-kb *Anomodon rugelii* genome (Fig 1, top) [13], was used as a comparison genome by Rice et al. [11] to elucidate the boundaries, gene content, and gene order of the moss DNA present in *Amborella*. Together, the four moss regions in *Amborella* add up to a 102-kb, nearly complete, *Anomodon-*type mitochondrial genome, with only four genes (all of them tRNA genes) completely missing from *Amborella* (see Δ3 and Δ4 in Fig 1, top). One of the four regions (Fig 1, bottom, MoAm4) has perfect synteny to sequenced moss genomes, while each of the other three regions (Fig 1, bottom, MoAm1-MoAm3) is composed of two perfectly syntenic segments joined together, making for a total of seven syntenic segments (labeled 1–7 in Fig 1). We refer to these seven segments as “moss-synteny segments”.

The nearly one-to-one gene correspondence and the strong similarities in size and synteny suggest that an entire mitochondrial genome from a single moss individual was transferred to *Amborella* [11]. However, this conflicts with the early phylogenetic evidence [7], described above, for three moss donors. To resolve this conflict we significantly increased sampling of moss taxa and of mitochondrial loci. Here we present this expanded phylogenetic analysis, which identifies two different moss donors, but does not support a third. We also develop a model for the creation of a chimeric moss genome from these two donors, as well as a detailed model for the subsequent recombination and rearrangement of this chimeric genome within the *Amborella* mitochondrial genome.

## Materials and Methods

Moss DNAs used in this study were provided by Jonathan Shaw’s lab at Duke University, Durham, North Carolina and by Ulfar Bergthorsson of the University of New Mexico, Albuquerque, New Mexico. See S1 Table for a list of these samples, including voucher numbers. In order to have enough material for multiple PCR reactions, moss DNAs were subjected to whole genome amplification using the RepliG mini kit (Qiagen) according to the manufacturer’s instructions. Primers for standard PCR were designed by hand from alignments of available bryophyte sequences. PCR products were purified by ExoSAP-IT or QIAquick gel extraction (GE Healthcare, Qiagen) and sequenced using the same primers used for PCR and an ABI (Applied Biosystem) instrument. Primers and low-quality sequence were removed manually, and base calls were checked using the program CodonCode Aligner (CodonCode Corporation). All sequences generated in this study are deposited in GenBank (see S1 Table for accession numbers XXXXXXX-YYYYYYYY).

All sequences were aligned by hand using the program Se-al (http://tree.bio.ed.ac.uk/software/seal). Maximum likelihood analysis was performed using all three codon positions and with gaps treated as missing characters using RaxML for all loci except locus P. RaxML was implemented using the general time reversible model with a gamma rate distribution and a random number seed equal to the alignment length. In order to reproduce the 2004 analysis [7], of *cox2*, and to examine the effects of expanded sampling, maximum likelihood analysis of locus P was conducted, as in the 2004 analysis, using GARLI 0.951 [16] with the general time reversible model and the following parameters: gamma distributed rate heterogeneity, four rate categories, and estimated proportion of invariant sites. In all but one case MODELTEST supported the GTR rate matrix and the gamma parameter settings. The single case of support for the HKY rate matrix was an unresolved locus (S1 Fig, locus O). Reanalysis in Garli using the HKY model [17] failed to resolve this locus (not shown). In all cases, five independent runs were used to ensure convergence on the best-fit tree and one thousand bootstrap replicates were performed.

Phylogenetic hypotheses were tested using the approximately unbiased (AU) test as implemented in the CONSEL software package [18], with AU *P*-values calculated from per-site likelihoods as determined by PAUP [19]. The AU *P*-values were based on the comparison of each unconstrained likelihood tree and a tree in which *Amborella* was constrained to group with the alternative group of putative donor mosses, i.e., when a given *Amborella* locus went with the Ptychomniales in the unconstrained tree, it was placed with (anywhere within or sister to) the Hookeriales in the constrained tree, and vice-versa.

## Results

### Phylogenetic analysis of moss donors to *Amborella* mtDNA

**Fig 2:**
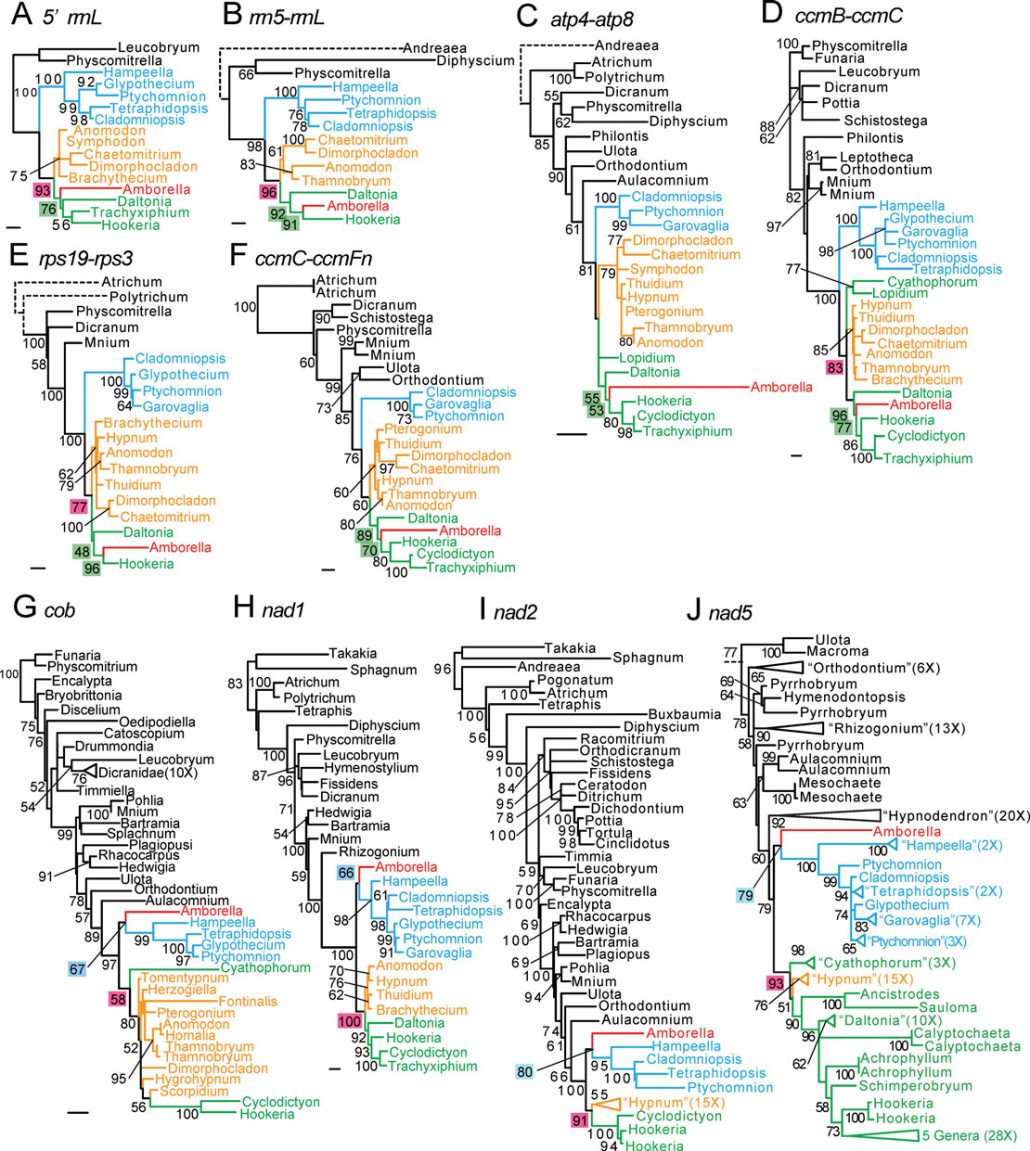
Phylogenetic evidence for two moss donors to *Amborella* mtDNA. These maximum likelihood trees were rooted based on the current best estimates of overall moss phylogeny [9,10,20–25]. The trees are labeled with shorthand names of the loci used in the analyses and with letters (A-J) that correspond to those in Figs 1 and 6 and Table 1. *Amborella* sequences are in red, Ptychomniales in blue, Hypnales in orange, and Hookeriales in green. Scale bars correspond to 0.01 substitutions per site. Bootstrap values >50% are shown, space permitting. Bootstrap values that support the monophyly of a clade comprising the Hypnales and Hookeriales are placed in light red boxes, those that support the placement of the *Amborella* sequences as sister to or within the Hookeriales are in green boxes, and those that support their placement as sister to the Ptychomniales are in blue boxes. Triangular branches indicate collapsed clades; these are given the name of a sampled genus belonging to the clade and a parenthetical number indicating how many taxa in the clade were sampled. In addition, the grade comprising the top part of tree J is not shown. For full sampling at these loci, see S1 Table. Dashed lines indicate branches whose lengths were reduced due to space constraints. Due to image size constraints only the relevant portion of a larger tree J is shown. The short stub of a dotted line indicating where tree J’s outgroup lineages were pruned.

**Fig 3:**
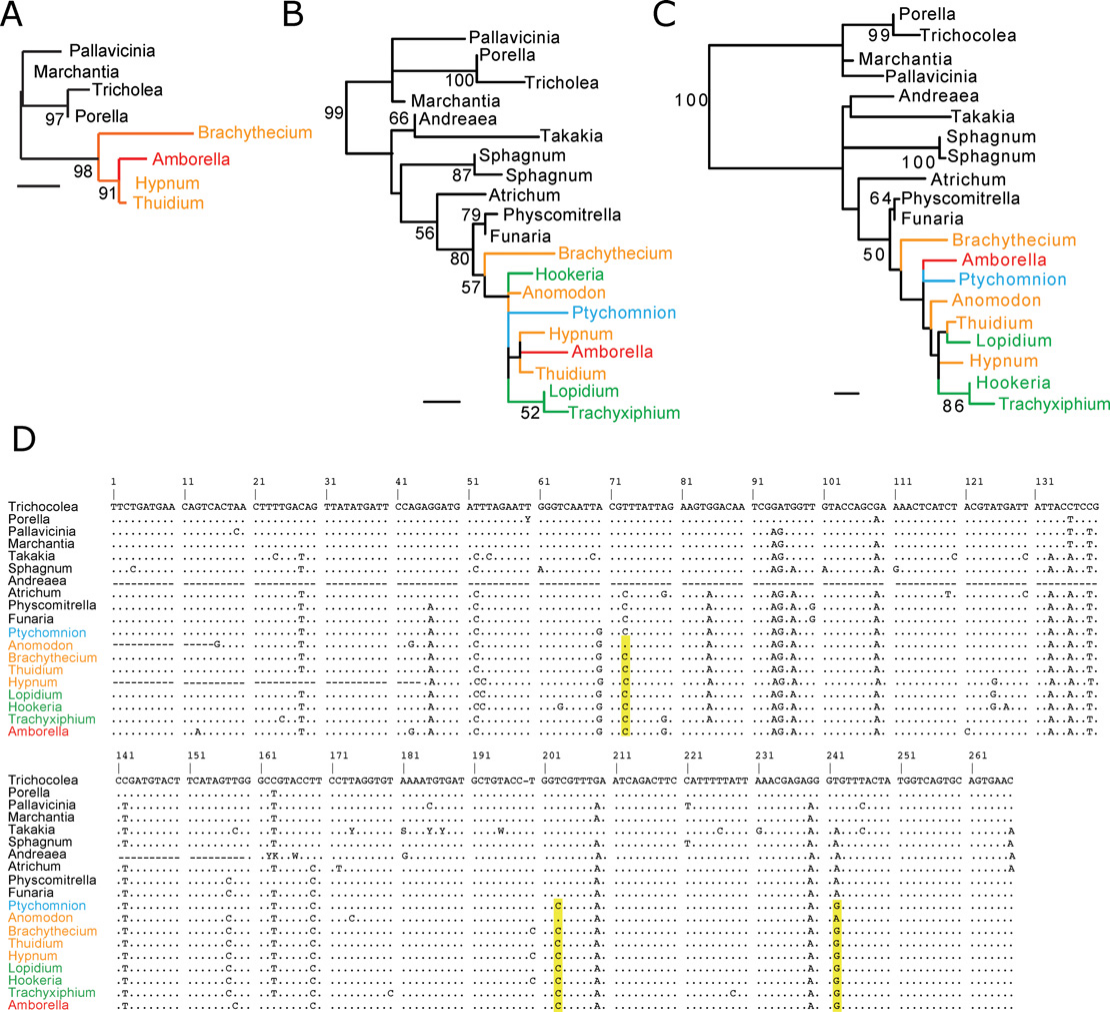
Updated analysis of *cox2* fails to support a Hypnales origin of this locus in *Amborella*. **A)** The bryophyte *cox2* tree from Fig 1 of [7]. *Amborella* sequences are in red, Hypnales in orange, and Hookeriales in green. Bootstrap values >50% are shown. Scale bars correspond to 0.01 substitutions per site. **B)** Expanded taxon sampling of the 266-bp *cox2* locus analyzed in A) and in [7]. **C)** Same taxon sampling as in B) but with the *cox2* alignment expanded to 881 bp. **D)** The *cox2* alignment used in B). Yellow highlights three characters responsible (see text) for the erroneous 2004 placement, as shown in A), of *Amborella cox2* within Hypnales.

A total of 16 mitochondrial loci (labeled A-P in Fig 1, top, and Table 1) were analyzed with maximum likelihood using data sets that contained between 6 and 166 moss sequences (Figs 2, 3 and S1 Fig), most of which were generated by PCR amplification as part of this study (S1 Table). The non-uniform representation of PCR amplified taxa across loci reflects the variable success of PCR amplification reactions at different loci. The seven moss-synteny segments in *Amborella* (Fig 1) were represented by between one and six of these 16 loci, which range in length from 266 to 2,383 bp. All well supported regions in these 16 trees were consistent with generally accepted relationships along the ‘backbone’ of moss phylogeny and of the major pleurocarp groups [9,10,20–25]. Thirteen of these trees supported either of two placements of the moss DNA in *Amborella*, within (or sister to) the Hookeriales or sister to the Ptychomniales). The other three trees were inconclusive owing to poor resolution of the three homocostate pleurocarp orders, missing taxa, and/or generally weak bootstrap support.

**Table 1:** Summary of phylogenetic analyses to identify moss donors to *Amborella*. NT = not tested.

Locus			Donor	AU test	
Name	Tree figure	Length (bp)		P-value	Significance
A	2A	902	Hookeriales	0.038	S
B	2B	820	Hookeriales	5.00E-04	S
C	2C	883	Hookeriales	0.251	NS
D	2D	758	Hookeriales	0.04	S
E	2E	1436	Hookeriales	0.062	NS
F	2F	905	Hookeriales	0.065	NS
G	2G	828	Ptych-sister	0.003	S
H	2H	1490	Ptych-sister	0.001	S
I	2I	2383	Ptych-sister	7.00E-05	S
J	2J	1157	Ptych-sister	0.089	NS
K	S1-K	775	Unresolved	NT	NT
L	S1-L	719	Unresolved	NT	NT
M	S1-M	941	Unresolved	NT	NT
N	S1-N	784	Ptych-sister	1.00E-04	S
O	S1-O	579	Unresolved	NT	NT
P	3A	266	Hypnales	NT	NT
P	3B	266	Unresolved	NT	NT
P	3C	881	Unresolved	NT	NT

Eight trees place the moss HGT sequences either within the Hookeriales (Fig 2B–2F), as sister to this order (Fig 2A), or of uncertain status with respect to these two placements owing to inadequate taxon sampling (S1L and S1M Fig). Six of these trees contain *Daltonia*, which, with the exception of the *Lopidium*-*Cyathophorum* clade, is the sister group to the rest of the sampled Hookeriales [9,10]. Five of these six trees (Fig 2B–2F) place the moss HGT sequence as sister to the rest of the Hookeriales to the exclusion of *Daltonia*, with bootstrap support of 91%, 53%, 77%, 96% and 70% respectively. In the sixth tree (Fig 2A), *Amborella* branches sister, with 76% support, to a weakly supported (34%) clade comprising *Daltonia* and the two other sampled Hookeriales. Finally, two loci (S1L and S1M Fig) place *Amborella* sister to the Hookeriales in analyses in which *Daltonia* sequences were not available. Taken together, these eight loci suggest that a substantial amount of the moss mtDNA in *Amborella* probably originated from within the Hookeriales

Five loci place *Amborella* as sister to the Ptychomniales, with 67%, 66%, 80%, 79%, and 41% bootstrap support (Fig 2G–2J and S1N Fig, respectively). For these loci, the Hookeriales + Hypnales clade is supported as monophyletic to the exclusion of *Amborella* with 58%, 100%, 91%, 93%, and 41% support, respectively.

We used the AU test [26] in the program CONSEL to test the significance of the 13 Hookeriales or Ptychomniales placements of *Amborella*-moss DNA. Six of these placements (three Hookeriales and four Ptychomniales) were significant at the p ≤ 0.05 level (Table 1).

**Fig 4:**
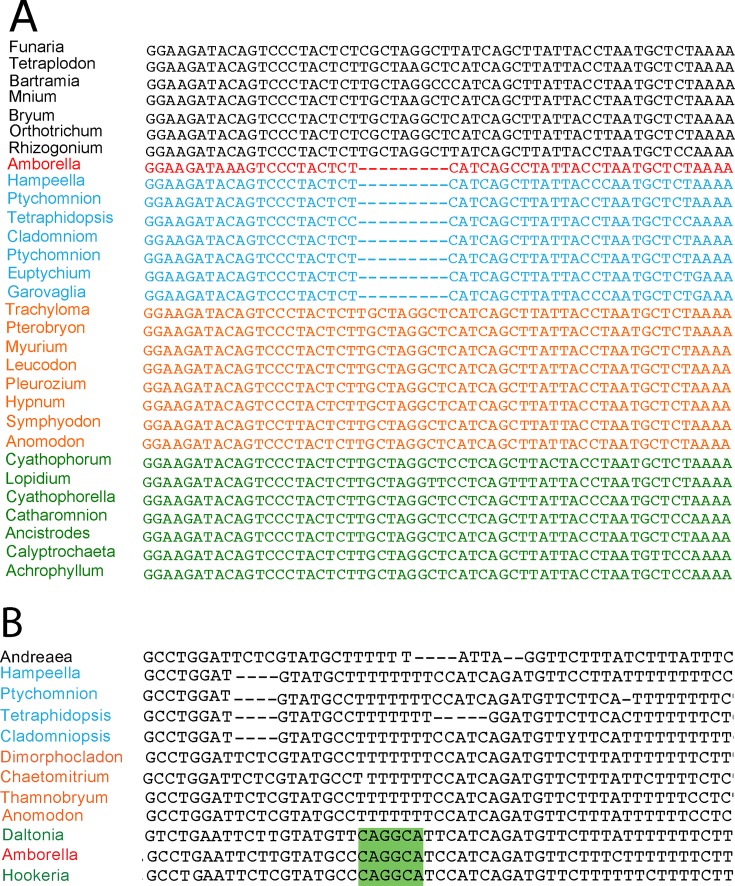
Two structural characters support a Ptychomniales-like (A) or Hookeriales (B) origin of portions of the moss mtDNA present in *Amborella*. Color scheme: Non-pleurocarps, black; *Amborella*, red; Ptychomniales, blue; Hypnales, orange; Hookeriales, green. Sequences within each color group are ordered phylogenetically. **A)** Part of the *nad5* intron alignment used for the phylogenetic analysis of Fig 2J, showing a 9-bp deletion uniquely shared by *Amborella* and all examined Ptychomniales. Note that there is no homoplasy for this indel region in the many taxa examined in Fig 2J that were excluded from the alignment shown here. **B)** Alignment of a portion of the *rrn5-*to-*rrnL* region used for Fig 2B. Highlighted is a 6-bp conversion tract (see text) uniquely shared by *Amborella* and both examined Hookeriales.

Two structural characters, whose regions were not included in the phylogenetic analyses, provide additional support for these two moss donors. A 9-bp deletion within intron 2 of *nad5* is uniquely shared by *Amborella* and all examined Ptychomniales (Fig 4A), in agreement with phylogenetic analysis of this locus (Fig 2J). Conversely, a 6-bp tract in the spacer between *rrn5 and rrnL* (locus B) groups *Amborella* with all examined Hookeriales (Fig 4B), in agreement with phylogenetic analysis of this locus (Fig 2B). The CAGGCA sequence that comprises this tract in *Amborella* and Hookeriales is part of a larger, 22-bp tract that matches the 5’ end of *rrnL* with only one mismatch in both *Anomodon* and *Amborella*. Given this similarity and that these two regions are only about 200 bp apart, a conversion event from the *rrnL* region to the CAGGCA site early in Hookeriales evolution seems likely.

None of the 16 loci tested supported an Hypnales donor, in contrast to the 2004 analysis of the moss-derived *cox2* gene of *Amborella* [7]. The 2004 analysis (reproduced in Fig 3A) placed *cox2* within the Hypnales (the only mosses then sampled) with 91% support. Reanalysis of the same, 266-bp *cox2* alignment used in 2004 with much better moss sampling failed to provide support for this placement (Fig 3B). Inspection of this expanded-taxon-sampling alignment reveals that the three characters (highlighted in yellow in Fig 3D) that were the basis of the 91% support for the 2004 placement of the *Amborella* sequence with two Hypnales to the exclusion of the third, *Brachythecium*, are the result of homoplasy in *Brachythecium*. At all three nucleotide positions, *Brachythecium* reverted to the ancestral character state subsequent to the relatively early evolution of the derived state, in the common ancestor of either the pleurocarps or the pleurocarps + outgroup mosses (Fig 3D). Analysis of the expanded taxon set shown in Fig 3B but with the *cox2* alignment increased to 881 bp (corresponding to locus P of Fig 2) also failed to provide meaningful support for a Hypnales origin of *Amborella cox2* (Fig 3C). We conclude that the 2004 *cox2* result [7] is a consequence of inadequate taxon sampling, the occurrence of three parallel reversals within a 266-bp portion of *cox2* in *Brachythecium*, and generally weak phylogenetic signal in this region.

### A chimeric moss mitochondrial genome in *Amborella*

Because moss mitochondrial genomes are so highly conserved (see [12–15] and Introduction), the Hookeriales and Ptychomniales-like donors to *Amborella* probably possessed identical mitochondrial gene orders and very similar genome sizes to those of the pleurocarp *Anomodon*. Accordingly, by mapping onto the *Anomodon* genome the four regions of moss DNA in *Amborella* (Fig 1, bottom) and the 13 phylogenetically diagnostic loci (Fig 1, top), we can estimate the number and sizes of the regions contributed by the two moss donors. Furthermore, the simplicity of the chimeric genome that emerged from this analysis allowed us to develop a two-step recombinational model for its origin.

The set of eight loci supporting a Hookeriales donor and the set of five supporting a Ptychomniales-like donor each circumscribes a single large, apparently donor-specific tract of DNA relative to *Anomodon* (Fig 1, top). This inference assumes that the region between each consecutive pair of loci from the same donor also derives from this donor. The Hookeriales tract comprises, at minimum, the 53 kb of DNA that extends from the left end of locus M to the right end of locus A (Fig 1, top). The Ptychomniales-like tract comprises, at minimum, the 32 kb of DNA that extends from the left end of locus J to the right end of locus G. Some 197 kb of moss-derived DNA is of uncertain origin; this DNA corresponds to the two regions located between the Hookeriales and Ptychomniales-like tracts (Fig 1, top).

**Fig 5:**
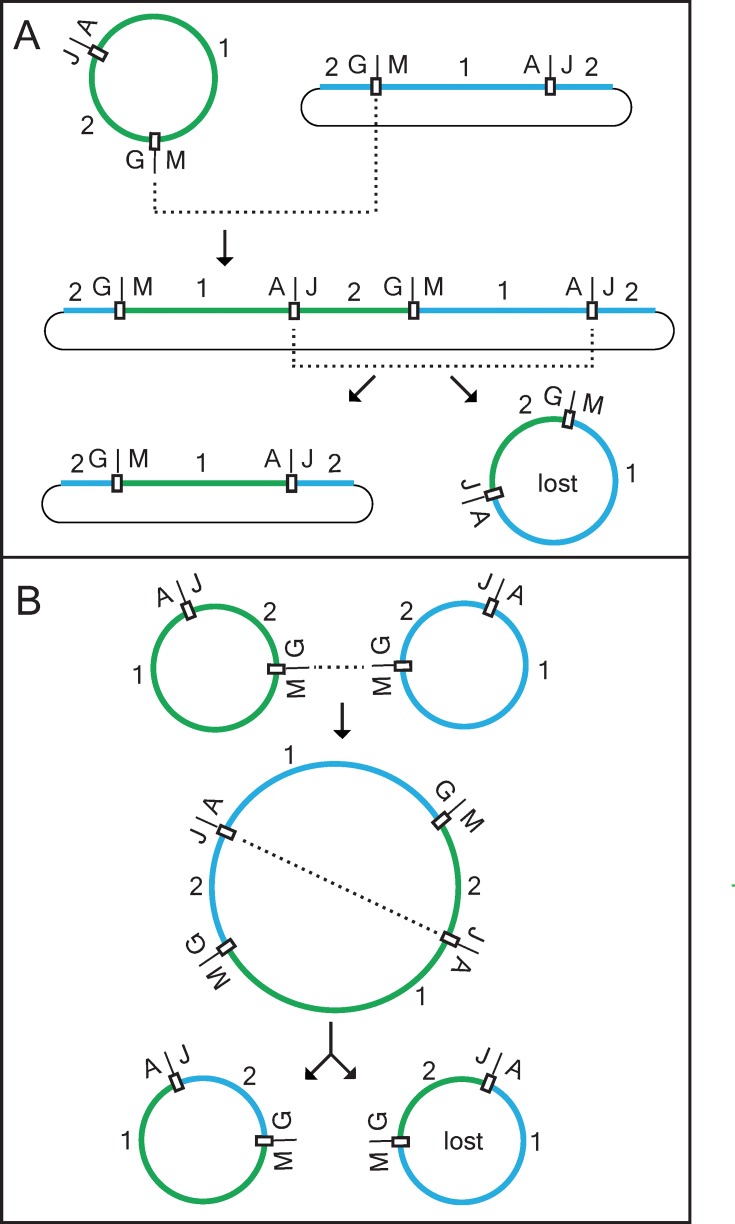
Two recombination models for the creation of a Ptychomniales/Hookeriales chimeric mitochondrial genome. These models differ only with respect to whether this chimeric moss genome is generated after (**A**) or before (**B**) integration of a moss genome into the *Amborella* mitochondrial genome. The nearly full-genome equivalent of moss mtDNA currently present in the *Amborella* mitochondrial genome (thin black lines) is divided into regions 1 and 2, with 1 corresponding to the roughly 60% of a moss genome-equivalent acquired from a member of the Hookeriales (thick green lines) and 2 the 40% acquired from a Ptychomniales-like donor (thick blue lines). Genomes are not shown to scale. Recombination sites are marked by open boxes. Recombination events are indicated by dotted lines. Both models show the same two recombination events, the first occurring intermolecularly between homologous moss G-M regions (see Fig 1, top) and the second intramolecularly between the A-J regions of a dimeric cointegrate moss genome that either is (**A**) or is not (**B**) already integrated within the *Amborella* genome. For what is meant by “lost”, see Results. To facilitate presentation and interpretation of these pathways, all molecules are shown as intact circular genomes. We recognize, however, that although plant mitochondrial genomes usually map/assemble as circular chromosomes, their *in vivo* conformation is probably a recombinationally dynamic population of circular and linear molecules of varying sizes [42,43].

Fig 5 shows two variants of a two-step recombination pathway that would create the chimeric moss genome shown in Fig 1 (top) via recombination of mitochondrial genomes belonging to members of the Hookeriales and a Ptychomniales-like group. These pathways assume horizontal transfer of two *entire* moss mitochondrial genomes. This is a sound assumption given the evidence 1) that plant mitochondrial HGT is driven by fusion of whole mitochondria from donor and recipient plants and 2) that *Amborella* mtDNA has acquired at least four entire mitochondrial genomes via HGT [11].

The two pathways involve the same two recombination events, differing only with respect to whether the chimeric moss genome is generated after or before integration of a moss genome into the *Amborella* mitochondrial genome. One pathway (Fig 5A) involves recombination between a circular moss genome (arbitrarily chosen to represent the Hookeriales donor) and a Ptychomniales-like genome that has integrated into the *Amborella* genome via a breakpoint arbitrarily selected to lie within the *cob* intron (see Fig 1, top, and next section). The other pathway (Fig 5B) involves recombination between circular genomes derived strictly from the two donor mosses.

In the first step in each pathway, the two moss genomes recombine at a homologous site located somewhere within either of the two regions corresponding to *Amborella* DNA of uncertain moss origin (Fig 5 arbitrarily shows this recombination as occurring between loci G and M; see Fig 1, top). This produces a chimeric head-to-tail dimer moss genome that is either integrated in the *Amborella* genome (Fig 5A) or not (Fig 5B). In the second step, recombination occurs somewhere within the other region corresponding to *Amborella* DNA of uncertain moss origin (the A-J region; see Fig 1, top). This event resolves each “dimer” into two smaller circular genomes, with both output genomes consisting entirely or partly of a whole-moss-genome equivalent of DNA that is a mixture of sequences of Hookeriales and Ptychomniales-like origin (Fig 5). The left output genome in each panel possesses the same, ca. 53/32 mixture of the two donor moss genomes as found in *Amborella*, whereas the right output genome possess the reverse proportion of donor sequences and was lost according to this model.

### Rearrangement analysis

**Fig 6:**
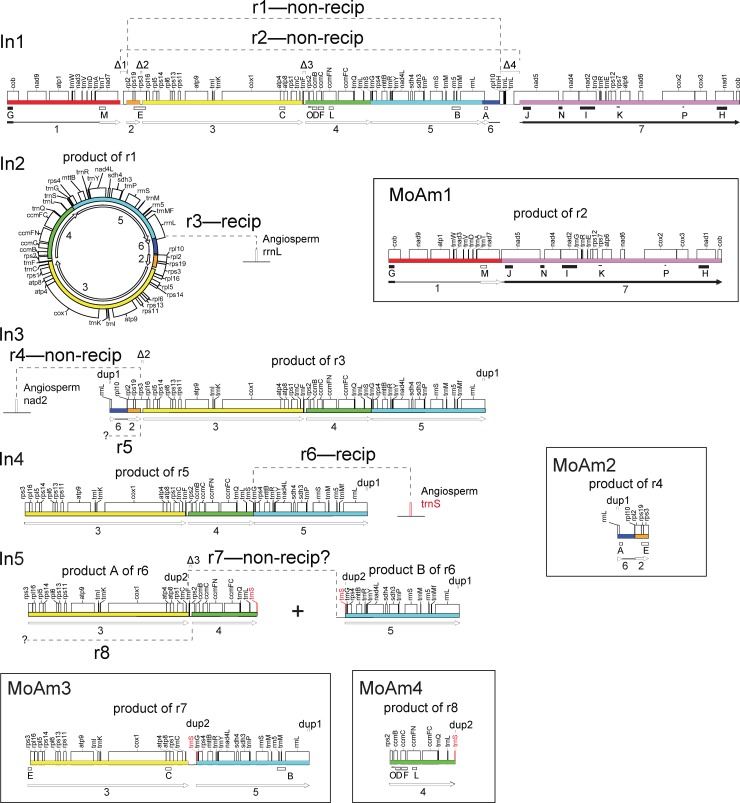
Model for the rearrangement of the moss mtDNA present in *Amborella*. **Shown are** a set of eight recombination events sufficient to produce the current organization and sequence content of the moss mtDNA present in the *Amborella* mitochondrial genome subsequent to its integration under the hypothesis of whole-moss-genome transfer. Note that this model is independent of the chimeric state of the donor moss “genome” and of models, such as those shown in Fig 5, for how this chimeric state arose. Note also that the order of r1-r8 as presented here is largely arbitrary (see Results). Intermediate stages of rearrangement are labeled In1-In5, while the final four products of rearrangement (i.e., the four regions of moss-derived DNA currently present in *Amborella* mtDNA) are boxed and labeled MoAm1-MoAm4. The top map (In1) shows (as in Fig 1, top) the mitochondrial genome of the reference moss *Anomodon* [13] arbitrarily linearized (see Results) within the *cob* intron to correspond to a donor moss genome immediately after its integration in *Amborella* mtDNA. Colored boxes and arrows indicate the position and relative orientation, respectively, of the seven blocks of synteny between the *Anomodon* genome and the four moss-derived regions in *Amborella*, with black arrows marking regions of Ptychomniales-like origin and open arrows marking regions of Hookeriales origin. The thin lines indicate the maximum extent of the three regions of unassigned origin. The 16 loci used for phylogenetic analysis are marked in In1 and in MoAm1-MoAm4 with rectangles or lines labeled A-P (see Fig 2 and S1 Fig and Table 1); a filled rectangle indicates a Ptychomniales-like origin, an open rectangle a Hookeriales origin, and a thin line an unresolved origin. The four deletions and two duplications >100 bp in length in *Amborella* relative to *Anomodon* are marked in selected intermediates and the four extant products by “Δ1-Δ4” and “dup1” and “dup2”, respectively.

We hypothesize that a full-length moss mitochondrial genome, either already chimeric or not, was inserted into the *Amborella* mitochondrial genome. This insertion could have occurred at any one of several recombination breakpoints depicted in Fig 6, which shows a model for the rearrangement of the moss DNA in *Amborella* subsequent to its integration. For the sake of diagrammatic simplicity, we arbitrarily chose an insertion breakpoint between the 80^th^ and 109^th^ bases of the *cob* intron, thus positioning the resulting portions of the *cob* gene at the termini of the horizontally acquired genome (Fig 1, top, and Fig 6, In1). Eight recombination events (r1-r8 in Fig 6) are sufficient to derive the current organization of the moss DNA in *Amborella* (Fig 1, bottom) from an intact, chimeric moss genome (represented by the Hookeriales and Ptychomniales-like tracts mapped onto the *Anomodon* reference genome in Fig 1, top) after its integration into the *Amborella* mitochondrial genome. These eight events account for not only the observed dispersion and synteny of the moss regions in *Amborella*, but also the six largest (>100 bp in length) deletions and duplications of the moss DNA in *Amborella* relative to *Anomodon*. The order of the eight rearrangement events as presented here is largely arbitrary and was chosen because it enables a convenient and straightforward model of rearrangement intermediates and recombination steps.

**Fig 7:**
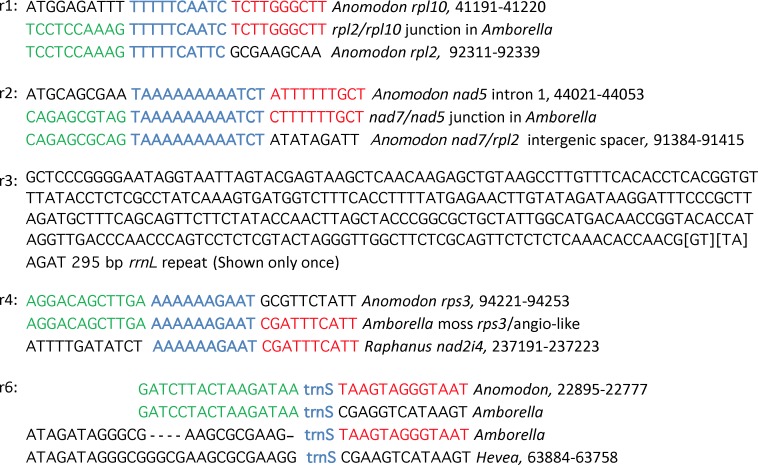
The five sets of repeats identified in rearrangement of the moss mtDNA present in *Amborella* mtDNA. For recombination events r1, r2, and 4, the very short repeats (10–13 bp in sizes) inferred to have mediated non-reciprocal recombination via the MMBIR pathway are in blue, while the left and right flanking sequences that are shared by two of the three extant examples of those repeats shown here are in green or red, respectively. Events r3 and r6 are reciprocal recombination events mediated by longer repeats, of either 84 bp (*trnS*) or 295 bp (a portion of *rrnL*), respectively.

Five of the eight rearrangements invoked under this model can be rationalized based on identified repeats (Fig 7 and below). Specifically, the predicted crossover products of certain repeats identified within *Anomodon*, or between *Anomodon* and non-moss regions in *Amborella* and/or other angiosperms, correspond precisely to what is observed at certain rearrangement junctions in *Amborella*. Three of these putatively repeat-mediated rearrangements occurred at very short repeats (10–13 bp in length). Studies of plant mtDNA recombination suggest that recombination at such short repeats takes place via a microhomology-mediated break-induced replication (MMBIR) mechanism [27,28] that leads to a duplicative, non-reciprocal cross-over event, i.e., only one of the two potential recombination products is produced, and the DNA substrate used for the recombination is preserved in its pre-recombination form. For example, if such recombination occurred across very short *direct* repeats, then either the circularized DNA between the repeats, or the union of the sequences flanking them, would be produced, but not both, and in either case the recombination substrate would be preserved. The other two inferred repeat-mediated rearrangements occurred non-duplicatively via reciprocal recombination across longer repeats (84 and 295 bp in length).

The first recombination event in this model (Fig 6, r1) occurred non-reciprocally between 10-bp direct repeats (Fig 7) located within moss genes *rpl2* and *rpl10* to form the circular product shown in Fig 6, In2. Recombination r2 also employed the entire chimeric moss integrate as substrate, taking place between 13-bp direct repeats (Fig 7) located in the *nad7*/*rpl2* intergenic spacer and the first intron of *nad5*. This nonreciprocal event, instead of circularizing the intervening stretch of DNA as with r1, formed a linear recombination product, i.e., it joined segments 1 and 7. This event formed one of the four extant moss-derived regions in *Amborella* (Fig 6, MoAm1). Subsequent to r1 and r2, the original chimeric moss integrate was lost, leaving *Amborella* mtDNA with the two rearrangement products shown in Fig 6, In2. This loss required no further recombination and presumably occurred by random sorting out. This is because, as initially produced, MoAm1 was functionally redundant to the original chimeric moss integrate, i.e., they contained the same set of non-moss sequences. Because the repeats responsible for r1 are closely nested within the boundaries of r2, the short regions between them, corresponding to Δ1 and Δ4 in Figs 1 and 6 (ln1), were also lost.

The 50-kb circular molecule from r1 was reintegrated via reciprocal recombination (r3) between its *rrnL* gene and an *rrnL* sequence (probably of angiosperm origin) located within the *Amborella* mitochondrial genome (Fig 6, In2). The extant moss integrant is terminated at both ends by a 295-bp *rrnL* repeat that is marked as “dup1” in Fig 6 (In3 and below).

Recombination r4 occurred non-reciprocally between 10-bp repeats (Fig 7) located within the moss *rps3* gene and an angiosperm *nad2* intron to create the extant region MoAm2 (Fig 6). Following this event, recombination r5, of unknown nature, separated the 6|2|3|4|5 substrate used for r4 into two regions, 3|4|5 (Fig 6, In4) and 6|2 (not shown). The 6|2 region was eventually lost, presumably by random sorting out. The loss of 6|2 also accounts for deletion Δ2 of the moss DNA in *Amborella* relative to the *Anomodon* reference genome (Fig 6, In1 and In3).

The *trnS* spanning the boundary of segments 4 and 5 recombined reciprocally (r6) with an angiosperm *trnS* elsewhere in the genome to create both intermediates shown in Fig 6, In5 (products A and B of r6). The DNA sequences currently flanking the duplicated *trnS* genes (“dup2”) at the terminus of segment 4 (Fig 6, MoAm4) and at the segment 3|5 boundary (Fig 6, MoAm3) are the predicted products of reciprocal crossover between a moss *trnS* and the *trnS*-UGA found in many angiosperms (Fig 7). *Amborella* mtDNA contains a horizontally transferred region that includes this angiosperm *trnS* context and which is identical to the *Hevea* sequence shown in Fig 7. Because the moss *trnS* recombined with an intact angiosperm *trnS* gene, an entire *trnS* gene is present in both segments 4 and 5 (Fig 6, see “dup2” in ln5, MoAm3, and MoAm4).

Recombination r7 is invoked to join segments 3 and 5 and thereby create the extant region MoAm3 (Figs 1 and 6), in which *trnC* (from segment 3) and *trnS* (segment 5) are now adjacent genes, separated by a 1702-bp spacer. Although good candidates for repeats mediating r7 could not be identified, the following evidence suggests that r7 may have occurred via non-reciprocal recombination. The 357-bp portion of the 1702-bp spacer adjacent to *trnC* is of moss mtDNA origin, corresponding almost precisely to the *trnC*/*trnF* spacer in *Anomodon* (see right end of segment 3 in Fig 6, In5). The remaining 1345 bp of this spacer is largely homologous to sequences present elsewhere in the *Amborella* mitochondrial genome, with the 96-bp portion immediately adjacent to *trnS* also adjacent to *trnS* in many angiosperm mtDNAs and thus part of the evidence for reciprocal recombination r6. Because the moss-derived 357-bp region is found *only* in moss mtDNAs, it seems unlikely that *Amborella* once possessed a sufficiently long copy of this region to recombine reciprocally with it. It is therefore more likely that r7 was a microhomology-based rearrangement event in which very short repeats at the ends of the moss-derived 357-bp spacer (Fig 6, In5, right end of segment 3) and the *Amborella*-derived 1345-bp spacer (Fig 6, In5, left end of product B) recombined in a non-reciprocal fashion.

Following r7, recombination r8 (of unknown nature) separated the segment 3 and 4 portions of the 3|4 substrate used for r7, thereby creating the extant region MoAm4 (Fig 6, bottom) and an isolated segment 3 (not shown). Segment 3 was eventually lost, again presumably by random sorting out, as was the segment 5 substrate used for r7. The loss of segment 3 also accounts for deletion Δ3 of the moss DNA in *Amborella* relative to the *Anomodon* reference genome (Fig 6, In1 and In5). In sum, the events described in this and the preceding five paragraphs suffice to create the four tracts of moss mtDNA (MoAm 1-MoAm4) that currently exist in the *Amborella* mitochondrial genome.

## Discussion

### At least two donors of the moss DNA in *Amborella*

Published structural analysis of the sequences comprising a virtually-complete moss mitochondrial genome located in the *Amborella* mitochondrial genome led to the conclusion that *Amborella* had incorporated an entire mitochondrial genome from a single moss donor [11]. In contrast, the phylogenetic analyses presented in this study establishes that the foreign moss DNA in *Amborella* was acquired from at least two moss donors, one belonging to the Hookeriales and the other to a lineage related to the Ptychomniales. The *cox2* evidence from an early PCR-based study [7] for a Hypnales donor disappeared with the improved taxon and character sampling of the present study.

The simplest interpretation of our phylogenetic data is that there were only two donor mosses, whose mitochondrial genomes recombined by as few as two steps (Fig 5) to create a chimeric genome composed of two long, contiguous tracts of Hookeriales and Ptychomniales-like sequence (Fig 1, top). However, these data are limited to 10 short loci that in aggregate cover only about 10% of a standard-sized moss mitochondrial genome (Fig 1, top). Accordingly, we cannot rule out the possibility of a more complex chimera, one with more intricate interspersion of Hookeriales- and Ptychomniales-derived sequences, and/or gene-converted regions acquired from as-yet unidentified moss donors, including multiple Hookeriales and/or Ptychomniales-like mosses that could not be resolved by the current analyses. These possibilities should be considered in light of: 1) the high frequency of gene conversion in angiosperm mtDNAs [29–35]; 2) the extravagant amount and diversity of HGT in *Amborella* [11]; 3) the unusually low rates of loss and rearrangement of large tracts of foreign mtDNA in *Amborella* [11]; and 4) the extreme conservation of moss mtDNAs in sequence content, synteny, size, and sequence similarity [13–15]. Once an entire mitochondrial genome from one moss was integrated into the *Amborella* genome, it would presumably serve as a very efficient landing pad (i.e., integration/conversion site) for potentially many more moss HGTs, as probably happened once already. Therefore, as more moss mitochondrial genomes are sequenced, especially from Hookeriales (no published or deposited genomes) and Ptychomniales (only one), it could be very interesting to re-explore the history of the moss DNA in *Amborella* using phylogenomic approaches.

### A three-genome merger–who merged with whom and when?

The moss mtDNA sequences present in the *Amborella* mitochondrial genomes represent the merger of at least three distinct and anciently divergent mitochondrial genomes, the *Amborella* genome itself and two moss genomes, one of Hookeriales ancestry and the other of unidentified ancestry but clearly related to the Ptychomniales. The two moss lineages last shared common ancestry with each other 150–200 million years ago and with *Amborella* over 450 million years ago [36,37]. The transfers of the two moss genomes to *Amborella* are thought to have occurred millions, possibly even tens of millions of years ago [11].

*Amborella* is endemic to the South Pacific island of New Caledonia. Among the ca. 520 species and 152 genera of mosses found in New Caledonia are members of three genera of Ptychomniales and 13 genera of Hookeriales [36,37,38]. Ptychomniales are found almost exclusively in several areas of the southern hemisphere (including New Caledonia), with highest generic diversity occurring in highland cloud forests, similar to those inhabited by *Amborella*. Hookeriales are more widespread but found principally in humid forests in the tropics and south temperate zone [37,38]. Biogeographic data are thus consistent with a member of each order having served as a donor of moss mtDNA to *Amborella*. How the moss-to-*Amborella* transfer(s) may have occurred is described in Rice et al., 2013 [11]. In brief, the authors postulated that the transfer(s) involved fusion of moss and *Amborella* mitochondria followed by whole-mitochondrial capture facilitated by epiphytic growth of mosses on *Amborella* and its propensity to produce new meristems/suckers at sites of wounding.

Three scenarios could account for this three-genome merger. One involves a moss-to-moss transfer (Fig 5B) that led to a lineage of mosses with a grossly chimeric but functional mitochondrial genome, one member of which then served as the only moss donor to *Amborella*. The second involves two successive moss-to-*Amborella* whole-genome transfers, from different donor lineages (Fig 5A). The third involves two simultaneous moss-to-*Amborella* whole-genome transfers. This scenario is consistent with both models shown in Fig 5, depending on whether the simultaneously captured moss genomes recombined with each before (Fig 5B) or after (Fig 5A) moss-genome recombination with the *Amborella* genome.

The only available evidence that in principle could decisively weigh in here is the extent of pseudogenization of the two phylogenetically distinct flavors of moss mtDNA present in *Amborella*. A pattern of consistently and significantly greater decay of Hookeriales-derived mtDNA in *Amborella* compared to Ptychomniales-like DNA (or vice-versa) would strongly favor scenario 2 over the other two. The two sources of moss DNA present in *Amborella* are, however, equally decayed, with the 17.0 kb of protein-coding DNA of Hookeriales descent possessing 26 pseudogene mutations (1.54 mutations/kb) and the 10.9 kb of Ptychomniales-like descent having 16 such mutations (1.46/kb) (these numbers are improved estimates that differ only slightly from those that can be derived from Table S2 of [11]). These results imply that the DNA from the two moss donors has been present in *Amborella* for roughly the same length of time. However, they fail to discriminate among the three transfer scenarios assuming that the two successive moss-to-*Amborella* transfer invoked under scenario 2 occurred relatively close in time.

Looking to the future, it is unlikely that evidence in favor of scenario 2 will ever be obtained, in the form of finding an *Amborella* relative that contains only one phylogenetic type of moss mtDNA or the other. This is because *Amborella trichopoda* is the only known survivor of a ca. 200-million-year-old lineage of angiosperms [39] and because its mother lode of foreign DNA was probably acquired in a common ancestor of all extant populations of the species [11]. In contrast, it is more likely that evidence in favor of scenario 1 would be obtained, in the form of the discovery of an extant Hookeriales moss whose mitochondrial genome is a roughly 3:2 chimera of native sequences and those acquired from a Ptychomniales-like moss, or vice-versa. This is because many extant mosses in the phylogenetic neighborhood of the moss donors (especially the Hookeriales) are available for genome sequencing. Finally, we fail to see any even-theoretical prospect for discovery of positive evidence for scenario 3 (two simultaneous moss-to-*Amborella* whole-genome transfers).

For three reasons, we favor scenario 2 (two successive moss-to-*Amborella* whole-genome transfers) as the most likely of these three scenarios. First, the extraordinary amount and diversity of foreign mtDNA present in *Amborella* mtDNA, including strong evidence for the capture of three entire green-algal mitochondrial genomes, makes the temporally separate acquisition of two moss mitochondrial genomes easy to imagine. Second, as noted in the preceding section, once one moss mitochondrial genome becomes integrated in the *Amborella* genome, it should serve as a very receptive site for integration, via homologous recombination, of a second moss genome. Third, the roughly 3:2 (or 2:3) mix of native to foreign moss mitochondrial sequences postulated to reside within a lineage of moss plants under scenario 1 requires functional compatibility between subunits of different origin for at least four key multi-subunit mitochondrial complexes: complexes I (*nad* genes) and IV (*cox* genes) of the electron-transfer chain, the ATP synthase, and the ribosome (Fig 1). This compatibility requirement would probably render such a grossly chimeric mitochondrial genome at least mildly deleterious relative to the native mitochondrial genome of the moss plant that served as recipient in a moss-to-moss transfer event.

### Mechanisms of moss mtDNA recombination in *Amborella*

Our models postulate a minimum of 11 recombination events to account for the number, synteny, and donors of the moss-derived regions present in *Amborella* mtDNA. Three of these events are “whole-genome” recombinations, the two shown in Fig 5 involving moss-to-moss recombination, plus the integration of a “moss genome” into the *Amborella* genome. The other eight recombinations are those within-*Amborella* events modeled in Fig 6.

Given the extreme conservation of moss mtDNAs with respect to size, synteny, and sequence similarity [12–15], the two moss-to-moss recombinations almost certainly occurred by reciprocal homologous recombination, presumably mediated by RecA. The moss-genome integration into the *Amborella* genome probably also occurred via reciprocal recombination, as a partial moss integrate would otherwise be a more likely outcome. Evidence presented in Results indicates that two within-*Amborella* rearrangements also occurred by reciprocal homologous recombination (Fig 6, r3 and r6. In contrast, three within-*Amborella* rearrangements (Fig 6, r1, r2, and r4) most likely occurred non-reciprocally at very short repeats via the MMBIR pathway, while r7 may also have occurred this way. This leaves two recombination events, r5 and r8, for which we have no evidence regarding underlying sequences and mechanism.

Evidence for reciprocal homologous recombination across large, usually perfect repeats in plant mtDNAs goes back three decades [40,41], with this process usually occurring at very high frequency and often generating genome assemblies consisting of a plethora of subgenomic and multimeric isomeric forms of the genome [42,43]. Only in recent years, however, has the importance of microhomology-mediated rearrangement (MHMR, i.e., recombination across very short repeats) as a major force in plant mtDNA reorganization become appreciated, including recognition that these events probably occur via MMBIR, a non-reciprocal and duplicative recombination mechanism [27,29]. MMBIR is an error-prone repair-backup pathway and possibly a mechanism to generate stress-induced variation that is normally suppressed in plants in deference to other mitochondrial repair systems. When genes involved in this suppression are knocked out in plants treated with a double-strand break inducer they accumulate MMHRs at high rates [44,45]. Such MHMR products have been found in wild-type plant mitochondria [46–51] and plastids [44].

It is uncertain whether the rearrangements of the moss mtDNA in *Amborella* were facilitated by a disruption of *Amborella*’s mitochondrial recombination and repair systems, or by a period of unusually high oxidative stress, or whether they represent the background level of MHMR in *Amborella* over millions of years. What is clear, however, is that the fragmentation of this foreign moss DNA provides a unique evolutionary window into the long-term rearrangement patterns and mechanisms in an angiosperm mitochondrial genome. For example, the three rearrangements for which MHMR-MMBIR is best supported all involve very short repeats that possess homopolymeric A/T stretches (Fig 7). Such sequences may preferentially act as MHMR sites owing to their relatively low melting temperature and tendency to cause replication-fork stalling, both of which could increase the likelihood of MMBIR [44]. Given the generally very high rate of rearrangements in angiosperm mtDNAs (*Amborella* actually appears to be anomalously retarded in this respect [11]), MHMR could be more instrumental in remolding the structure of these genomes than is currently appreciated.

An important implication of MHMR for our model of evolutionary recombination and rearrangement is that all MHMR models yield non-reciprocal crossover products, so that recombination between two copies of repeat R, flanked by unique sequences A + B or C + D, produces, for example, A-R-D but not C-R-B. The non-reciprocal nature of these events seems especially relevant considering the overall lack of duplications and deletions of the moss-derived sequence in *Amborella*. For example, under our model the two largest deletions (Figs 1 and 6, Δ1 and Δ4) result from the fixation of the products of two independent non-reciprocal crossovers (r1 and r2) whose recombining short repeats happen to be located near each other. Even more striking are events r4 and r5, and also r7 and r8, whose underlying repeats, either identified or not, are even closer together, resulting in deletions of only 314 bp (Δ2) and 436 bp (Δ3) in length, respectively. Of all the possible combinations of repeat pairs, why are these, which result in so little deletion, observed? One possibility is that they are not independent. Perhaps a recombination event at one site greatly increases the probability of concomitant recombination at a nearby site because the act of recombination exposes the DNA region encompassing both sites to the recombination machinery and, perhaps, because recombination is to some extent processive.

### Envoi

Our findings reveal that *Amborella* mtDNA may have acquired an entire moss mitochondrial genome via HGT not just once [11] but twice. This would make a total of five whole-genome transfers, two from mosses and three from green algae, in the history of the astonishing *Amborella* mitochondrial genome. The large amount of foreign angiosperm mtDNA present in the *Amborella* genome raises the specter of even more whole-genome transfers, in particular, from parasitic plants in the Santalales [11]. However, the very large and variable sizes and rapidly rearranging nature of angiosperm mtDNAs render reconstruction of such potential transfers extremely challenging. It is precisely the opposite evolutionary dynamics of moss mtDNAs–small, highly conserved in size, and absolutely conserved in synteny–that, together with unusually low rates of mtDNA rearrangement in *Amborella* [11], enabled the original detection of what seemed to be a single whole-genome transfer from mosses. In conjunction with the phylogenetic analyses reported herein, these dynamics have facilitated the present inference of two such transfers and the detailed reconstruction of a complex set of subsequent recombination events and, in some cases, underlying sequences and mechanisms. These events first created an intact but chimeric moss genome within *Amborella* mtDNA and then fragmented and rearranged this genome into the four blocks and seven syntenic regions of moss DNA now present in *Amborella*. The *Amborella* mitochondrial genome is a veritable marvel in having captured and kept multiple entire foreign genomes, with “the” chimeric moss genome providing unexpected insight into the fate of one such genome in the context of neutral evolution.

## Supporting Information

S1 Fig Phylogenetic analysis of five of the 16 loci used to examine the origin of the moss mtDNA present in *Amborella* (the other 11 analyses are shown in Figs 2 and 3). These maximum likelihood trees were rooted based on the current best estimates of overall moss phylogeny [9,10,20–25]. The trees are labeled with shorthand names of the loci used in the analyses and with large letters (K-O) that correspond to those in Figs 1 and 6 and in Table 1. *Amborella* sequences are in red, Ptychomniales in blue, Hypnales in orange, and Hookeriales in green. Bootstrap values that support the monophyly of a clade comprising the Hypnales and Hookeriales are placed in light red boxes, those that support the placement of the *Amborella* sequences as sister to or within the Hookeriales are in green boxes, and those that support their placement as sister to the Ptychomniales are in blue boxes. Scale bars correspond to 0.01 substitutions per site. Bootstrap values >50% are shown, except that three key (i.e., color boxed) values ≤50% are also given. The dashed line indicates a branch whose length was reduced due to space constraints.
(TIFF)

S1 Table Accession numbers for the sequences used in this study. (XLSX)
