# Supplementary figures and images for: The Complete Moss Mitochondrial Genome in the Angiosperm Amborella Is a Chimera Derived from Two Moss Whole-Genome Transfers
Source: PLoS One. 2015 Nov 30;10(11):e0137532. doi: 10.1371/journal.pone.0137532 (PMC4664403; doi:10.1371/journal.pone.0137532)

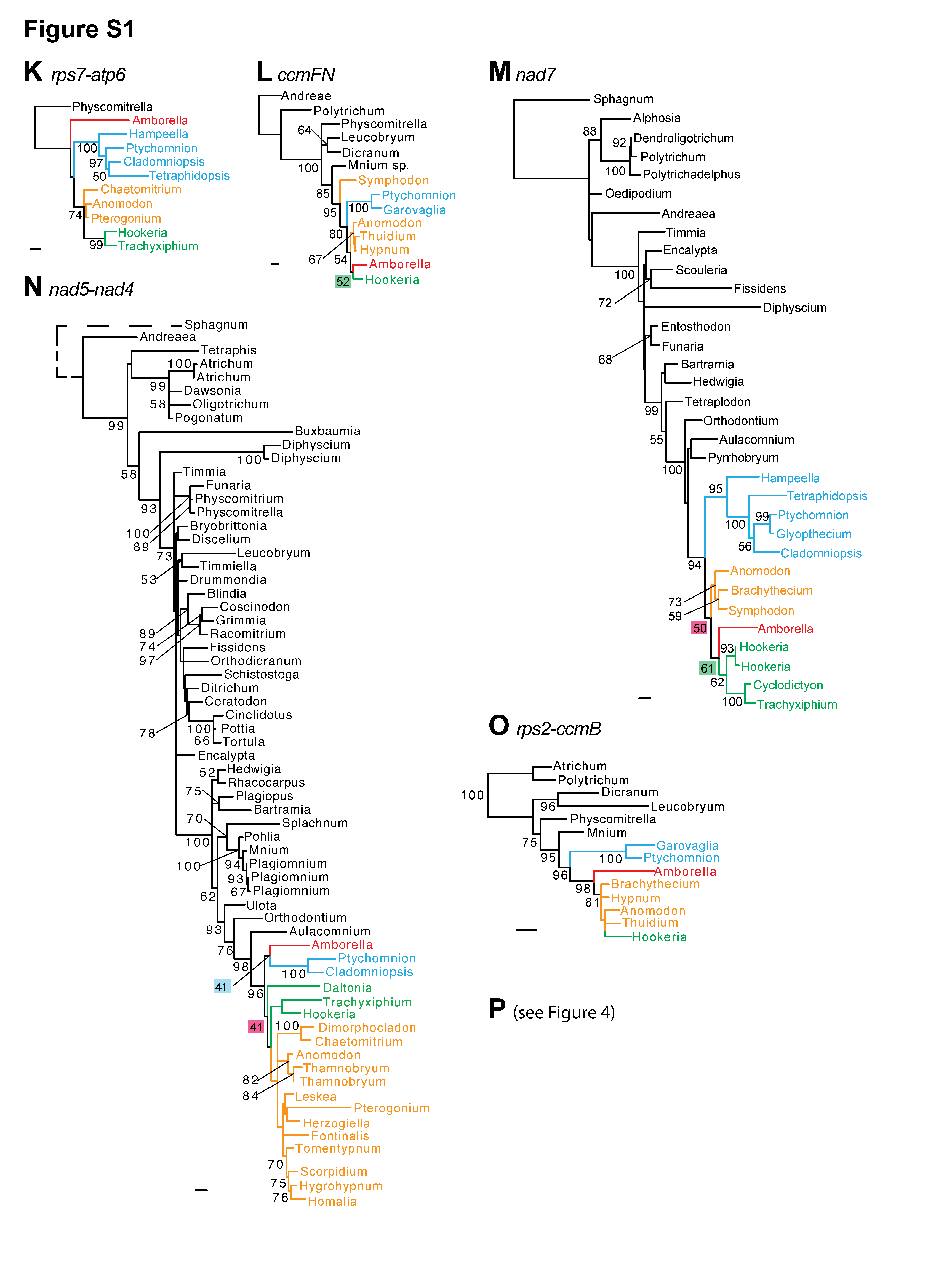

Supplement: S1 Fig — These maximum likelihood trees were rooted based on the current best estimates of overall moss phylogeny [9,10,20–25]. The trees are labeled with shorthand names of the loci used in the analyses and with large letters (K-O) that correspond to those in Figs 1 and 6 and in Table 1. Amborella sequences are in red, Ptychomniales in blue, Hypnales in orange, and Hookeriales in green. Bootstrap values that support the monophyly of a clade comprising the Hypnales and Hookeriales are placed in light red boxes, those that support the placement of the Amborella sequences as sister to or within the Hookeriales are in green boxes, and those that support their placement as sister to the Ptychomniales are in blue boxes. Scale bars correspond to 0.01 substitutions per site. Bootstrap values >50% are shown, except that three key (i.e., color boxed) values ≤50% are also given. The dashed line indicates a branch whose length was reduced due to space constraints. (TIFF) [file pone.0137532.s001.tiff]
